# Nematodynamics with odd and rotational viscosities

**DOI:** 10.1140/epje/s10189-024-00441-8

**Published:** 2024-07-27

**Authors:** L. M. Pismen

**Affiliations:** Department of Chemical Engineering, Technion – Israel Institute of Technology, 32000 Haifa, Israel

## Abstract

We explore a novel mechanism of interactions between nematic order and flow including odd and rotational viscosities, and investigate activity-induced instabilities in the framework of this model. We show how these modes of viscous dissipation can be incorporated in the Ericksen–Leslie formalism, but it does not eliminate deficiencies of the approach based on Onsager’s reciprocal relations that lead to spurious instabilities. The suggested way of deriving nematodynamic equations, based on a specific mechanism applicable to rigid rods, is not universal, but it avoids referring to Onsager’s relations and avoids spurious instabilities in the absence of an active inputs. The model is further applied to the analysis of instabilities in active media

## Introduction

The renewed interest in dynamics of nematic fluids has arisen owing to its wide applications in studies of active matter. Nematic order is commonly encountered in biological tissues [1–3], cells [4, 5], and bacterial swarms [6, 7] colonies [8], and biofilms [9]. In collectives of rodlike particles [10–12] and biopolymers [13–15], nematic orientation is combined with polar activity. In all these systems, the constitutive elementary units are macroscopic, in contrast to molecular units of common nematic liquids. Nevertheless, theory of active nematic is commonly based on the established nematodynamic theory. The original Ericksen–Leslie approach [16–18] employing the nematic director as the order parameter, has been later extended to allow for a variable modulus through the use of the tensor order parameter [26, 27]. Although nematic active matter evolves far from equilibrium, its theory is commonly based on theory of passive nematics supplemented by a phenomenological active input.

The established theories aspire to be universal: they do not assume any specific mechanism of interactions between rearrangements of nematic order and flow, but derive dynamics close to equilibrium by assigning general linear relationships between thermodynamic forces and fluxes and establish relations between their coefficients via Onsager’s reciprocal relations. There are general objections to this approach [28] on the basis that a combination of variables even and odd under time reversal is “physically unsound”, and cannot ensure evolution to thermodynamic equilibrium. Indeed, it has been recently shown by straightforward computation [29] that instability may arise in the Ericksen–Leslie equations even in the absence of an active input, when it is ostensibly forbidden by Onsager’s relations.

This paper suggests a simple specific mechanism of interaction between nematic order and flow, which is most likely to be applicable to systems with macroscopic rodlike elementary units. This mechanism generates viscous anisotropy involving both *rotational* and *odd* viscosity. The latter has been introduced in the context of superfluidity and quantum Hall effect [19] and otherwise is known to be present either in chiral media [20, 21] or in fluids containing spinning components, see [22, 23], as well as the review [24] and references therein. We explore how these effects modify the Ericksen–Leslie approach, but, of course, they cannot eliminate spurious instabilities. We continue with extending the model to accommodate a variable modulus.

## Friction effects in a passive nematic

### Antisymmetric part of the viscous tensor

Rather than describing the hydrodynamic effect of nematic rearrangement in the standard roundabout way based on Onsager’s reciprocity relations, we derive nematodynamic equations directly by considering the mechanisms of both flow effect on local orientation and frictional dissipation (additional to that due to mean flow) caused by translation and rotation of oriented particles relative to the prevailing average velocity.

A velocity gradient along a rigid (rodlike) oriented particle $(\textbf{n} \cdot \nabla )\textbf{v}$ causes the director $\textbf{n}$ to rotate with the angular velocity

1 $$\begin{aligned} \omega _a=\varepsilon _{abk} n_b n_l \partial _l v_k \;\; \mathrm {in \;3D}, \;\; \omega =\varepsilon _{bk} n_b n_l\partial _l v_k\;\; \mathrm {in \; 2D}. \end{aligned}$$

Here and further on, summation over repeating indices is implied, and 2D expressions are reduced from 3D by dropping the index *a*. Rotation is counteracted by the effective friction force per unit volume $f_a$ linear in $\omega _a$, which can be transformed in 3D to the stress tensor $\sigma _{ij} =\varepsilon _{ija}f_a$. The explicit 3D form (excluding so far viscous coefficients) is

2 $$\begin{aligned} \sigma _{ij}=\varepsilon _{ija}\varepsilon _{abk} n_b n_l s_{kl}, \end{aligned}$$

where $s_{kl}=\partial _k v_l$ is the strain tensor, which is commonly separated into symmetric and antisymmetric (vorticity) parts, $s^\pm _{ij}=\textstyle \frac{1}{2}(s_{ij}\pm s_{ji})$. The resulting viscous tensor $\eta _{ijkl}$ in the stress–strain relation $\sigma _{ij} =\eta _{ijkl}s_{kl}$ has a general form (excluding so far relevant coefficients)

3 $$\begin{aligned} \eta _{ijkl} = \varepsilon _{ija} \varepsilon _{abk} n_b n_l. \end{aligned}$$

This fourth-order tensor is antisymmetric in *i*, *j* but has no definite symmetry in *k*, *l*. It can be split into symmetric antisymmetric parts, $\eta _{ijkl}^\pm =\textstyle \frac{1}{2}(\eta _{ijkl}\pm \eta _{ijlk})$, which can be assigned distinct viscosities $\eta _\pm$:

4 $$\begin{aligned} \eta _{ijkl}^\pm =\textstyle \frac{1}{2} \eta _\pm \varepsilon _{ija} (\varepsilon _{abk} n_b n_l \pm \varepsilon _{abl} n_b n_k) . \end{aligned}$$

According to the general classification of viscous tensors (Table 1 in Ref. [25]), the coefficient $\eta _- \equiv \eta _\textrm{r}$ in the tensor $\eta _{ijkl}^-$ odd in both pairs of indices *i*, *j* and *k*, *l* is identified as the *rotational* viscosity, and the coefficient $\eta _+ \equiv \eta _\textrm{o}$ in the tensor $\eta _{ijkl}^+$ odd in *i*, *j* but even in *k*, *l*, as *odd* viscosity. These anisotropic terms supplement the common isotropic viscosity tensor $\eta _{ijkl}^s=\eta _s\delta _{ik}\delta _{jl}$.

### Linear stability analysis

Due to director’s rotation, flow is also affected by the change of elastic energy per unit volume, defined by an appropriate Landau–de Gennes Lagrangian $\mathcal {L}$. The molecular field $\textbf{h}$ resulting from virtual orientational distortions is defined in the standard way [18] as

5 $$\begin{aligned} h_j =\partial \mathcal {L}/\partial n_j + \partial _i \pi _{ij}, \qquad \pi _{ij}= \partial \mathcal {L} /( \partial _in_j), \end{aligned}$$

which generates the distortion stress tensor $\sigma _{ij}^\textrm{d}= -\pi _{ik}\partial _jn_k$. Combining all these contributions brings the Navier–Stokes equation to the general form

6 $$\begin{aligned} \rho \mathcal {D}v_i= \partial _j (\sigma ^\textrm{s}_{ij}+\sigma ^\textrm{a}_{ij}+\sigma ^\textrm{d}_{ij}) - \partial _i p . \end{aligned}$$

where the stress tensor $\varvec{\sigma }^\textrm{a}=\varvec{\sigma }^\textrm{o}+\varvec{\sigma }^\textrm{r}$ antisymmetric in *i*, *j* combines the odd and rotational stresses; $\mathcal {D}=\partial _t + \textbf{v}\cdot \nabla$ is the substantial derivative, $\rho$ is density and *p* is pressure.

For the purpose of linear stability analysis of Eq. (6) to $\mathcal {O}(\epsilon )$ perturbations of the ordered state in 2D oriented, say, along the axis $x_1$, it is advantageous to express the perturbation velocity $\widetilde{\textbf{v}}$ through the stream function, $\widetilde{v}_i =\varepsilon _{ij}\partial _j\varPsi$, and expand the latter as $\varPsi =\epsilon \int \widehat{\varPsi }(\textbf{k})\textrm{e}^{\textrm{i}\mathbf { k\cdot x}}\textrm{d}\textbf{k}$, where $\textbf{k}$ is a perturbation wave vector. With the base state $n_1^0=1, \,n_2^0=0$, the indices *b*, *l* in Eq. (3) should be set to 1. Hence, the only $\mathcal {O}(1)$ elements of this tensor are $\eta _{ij21} =\varepsilon _{ij}\eta ^\textrm{a}$ with $\eta ^\textrm{a} =\textstyle \frac{1}{2}(\eta ^\textrm{o}+\eta ^\textrm{r})$, so that $\sigma ^\textrm{a}_{ij}= \varepsilon _{ij}\eta ^\textrm{a}s_{12}$, Fourier-transformed as $-\varepsilon _{ij}k^2\eta ^\textrm{a}\varPsi \sin ^2\varphi$, where $\varphi$ is the angle between the perturbation direction and the unperturbed orientation.

For an incompressible fluid, taking the curl of Eq. (6) eliminates pressure. This brings the Fourier transform of Eq. (6) to

7 $$\begin{aligned} \rho \mathcal {D}\widehat{\varPsi }= -k^2 \left( \eta _\textrm{s} +\eta _\textrm{a} \sin ^2\varphi \right) \widehat{\varPsi }. \end{aligned}$$

The distortion stress vanishes upon linearisation, so that the influence of nematic alignment on hydrodynamics is restricted to $\mathcal {O}(\epsilon ^2)$, and linear stability of Eq. (6) is evident. The dynamic equation of $\widetilde{\textbf{n}}$ obtained by varying the Lagrangian is stable as well.

### Modified Ericksen–Leslie approach

We explore now how adding odd and rotational viscosities would modify the traditional approach of the Ericksen–Leslie nematodynamic theory [16–18], which couples changes of nematic orientation and fluid motion by tracing the change of entropy in the course of evolution and requiring the resulting equations to satisfy Onsager’s reciprocity relations. This subsection is purely illustrative, since odd viscosity implies broken time-reversal symmetry of the system [19], and hence, we do not expect Onsager’s relations to be applicable. However, the standard approach also involves a term odd under time reversal, $\textbf{N}= \partial _t \textbf{n}-\textbf{s}^- \cdot \textbf{n}$, which, nevertheless, did not disqualify this approach, still considered to be standard.

The standard theory starts from the equation of entropy change

8 $$\begin{aligned} T\partial _t S= \int \left( \sigma _{ij}s_{ij}+\mathbf {h\cdot N}\right) \textrm{d}\textbf{x}, \end{aligned}$$

With a commonly used symmetric viscous tensor, (8) depends on the symmetric strain only, but odd and rotational viscosity tensors antisymmetric in *i*, *j* necessitate adding here the antisymmetric strain as well, so that the total change of entropy is presented as

9 $$\begin{aligned} T\partial _t S= \int \left( \sigma _{ij}^\textrm{s} s^+_{ij}+\sigma _{ij}^\textrm{a} s^-_{ij} +\mathbf {h\cdot N}\right) \textrm{d}\textbf{x}, \end{aligned}$$

According to the standard procedure, the contributions to the entropy source are presented as products of fluxes by conjugate forces with coefficients having the dimension of viscosity. In the standard formalism, $\textbf{h}$ is identified as the force conjugate to $\textbf{N}$ and $\sigma _{ij}^\textrm{s}$ as the force conjugate to $s^+_{ij}$. Including antisymmetric viscous stresses brings about an additional pair: $\sigma _{ij}^\textrm{a}$ as the force conjugate to $s^-_{ij}$. The forces are tied to the fluxes by linear relations with coefficients having the dimension of viscosity arranged into tensors of an appropriate rank, which, by the Onsager principle, should be symmetric to interchange of forces and fluxes. The amended relations take the general form

10 $$\begin{aligned} \sigma _{ij}^\textrm{s}&=S^+_{ijkl} s^+_{kl}+B_{ijkl}s^-_{kl}+G^+_{ijk}N_k, \end{aligned}$$

11 $$\begin{aligned} \sigma _{kl}^\textrm{a}&=B_{ijkl}s^+_{ij}+S^-_{ijkl}s^-_{ij}+G^-_{kli}N_i, \end{aligned}$$

12 $$\begin{aligned} h_k&=G^+_{ijk}s^+_{ij}+G^-_{ijk}s^-_{ij}+ G_{ki} N_i. \end{aligned}$$

Since the structure of the matrices in Eq. (12) must be compatible with the local symmetry of the nematic, they must contain only the director $\textbf{n}$ and be symmetric to its reversal. Thus, the matrices $\textbf{S}^\pm$ and $\textbf{B}^\pm$ must contain an even number of $\textbf{n}$’s. Since $\textbf{h}$ and $\textbf{N}$ are odd while $\textbf{s}$ and $\varvec{\sigma }^\textrm{s}$ are even under reversal of $\textbf{n}$, the matrix $\textbf{G}^\pm$ should contain at least a single $\textbf{n}$. The matrix $S^+_{ijkl}$ is symmetric to the transposition of the two pairs of indices *i*, *j* and *k*, *l*, while $S^-_{ijkl}$ is antisymmetric in *i*, *j* but has no symmetry to the transposition of *k*, *l*, since odd and rotational viscosities are not separated here. The matrix $B_{ijkl}$ is symmetric to the transposition of the first and antisymmetric to the transposition of the second pair of indices. The matrix $G^+_{ijk}$ is symmetric and $G^-_{ijk}$ is antisymmetric to the transposition of the first two indices.

To avoid proliferation of parameters, we explore a simplified version with anisotropy retained only in the matrix $S^-$ to comply with Eq. (3). With $B_{ijkl}=\beta \delta _{ij}\varepsilon _{kl}$, the contribution of the isotropic part of $\varvec{\sigma }^\textrm{s}$ to $\varvec{\sigma }^\textrm{a}$ vanishes, but is restored by adding the anisotropic part $\beta ' n_in_j\delta _{ij}\varepsilon _{kl}$. Other tensors are simplified as $G^+_{ijk}=\gamma _+\delta _{ij}n_k, \,G^-_{ijk}=\gamma _-\varepsilon _{ij}n_k, \, G_{ki}=\gamma \delta _{ki}$. The dynamic equation of the director follows from Eq. (12):

13 $$\begin{aligned} \partial _t n_i = (s^-_{ij}- \chi _+ s^+_{ij}- \chi _-s^-_{ij}) n_j +\varGamma \,h_i. \end{aligned}$$

where the mobility coefficient is $\varGamma =\gamma ^{-1}$, and the alignment parameters $\chi _\pm = \gamma _\pm /\gamma$ determine the response of the director to local shear and rotation. Collecting all components of the total stress leads to the enhanced nematic flow equation

14 $$\begin{aligned} \rho \partial _tv_i= \partial _j (\sigma ^\textrm{s}_{ij}+\sigma ^\textrm{a}_{ij}+\sigma ^\textrm{p}_{ij}) - \partial _i p . \end{aligned}$$

where $\varvec{\sigma }^\textrm{p}$ is the part of the total stress related to nematic alignment, usually called passive stress in the theory of active nematics, which includes both the distortion stress and the interaction terms from Eq. (12):

15 $$\begin{aligned} \sigma ^\textrm{p}_{ij}=\textstyle \frac{1}{2} \chi _+ (n_ih_j+h_in_j)+\textstyle \frac{1}{2}\chi _-(n_ih_j-h_in_j) + \sigma ^\textrm{d}_{ij} . \nonumber \\ \end{aligned}$$

The time derivative in Eqs. (13), (14) can be replaced by the substantial derivative $\mathcal {D}$ as in Eq. (6).

Instabilities, originating in the terms in Eq. (15) additional to $\varvec{\sigma }^\textrm{d}$ introduced trough Onsager’s relations have been shown to occur in the absence of asymmetric viscous stress [29, 30]. There is no chance that the described enhancements, which introduce additional parameters but lack strong stabilising factors, would be able to counteract the instability under all circumstances. This, in effect, invalidates the entire standard theory, as spontaneous deviations from equilibrium are impossible in the absence of either internal or external energy sources.

## Instabilities in an active system

### Instabilities in the director-based description

Evidently, instabilities may only arise in active systems. Activity is commonly introduced as an active stress $\sigma _{ij}^\textrm{z}=-\zeta n_in_j$ with the parameter $\zeta$ positive for extensile and negative for contractile activity. This adds to the Navier–Stokes equation (6) an orientation-dependent term, and coupling between nematic alignment and flow may be now destabilising. As an illustration, we consider linear stability of an ordered state. The unperturbed alignment direction is irrelevant, and can be chosen along the $x_1$ coordinate. Due to the rotational symmetry, 2D formulation is sufficient, so that the base state is $n_1^0=1, \,n_2^0=0$. Upon linearisation and applying the operator $\varepsilon _{il}\partial _j\partial _l$, the active stress supplements the Fourier-transformed hydrodynamic equation (7) by the term

16 $$\begin{aligned} \zeta k^2 (\widehat{n}_1 \sin 2\varphi -\widehat{n}_2 \cos 2\varphi ). \end{aligned}$$

The evolution equation of the nematic director derived by varying the energy functional is supplemented by the local rotational term $\varepsilon _{ij}\omega n_j$ with $\omega$ given by Eq. (1). It also retains the global rotational term $s^-_{ij}n_j$, which takes into account that the energy does not change when both the nematic alignment and the fluid as a whole are rotated with the same angular velocity. Thus, advection-alignment coupling is retained without relying on Onsager’s principle, with the antisymmetric viscosity stress being responsible for rotational anisotropy. This input, specific to the interaction mechanism described in Sect. 2.1, governs the flow alignment of the director, replacing the alignment parameter that appears in the Ericksen–Leslie theory, but it is not accompanied by reverse coupling generating spurious instabilities [29]. Another substantial difference is the absence of a symmetric convective term $s^+_{ij}n_j$. The 2D equation reads

17 $$\begin{aligned} \mathcal {D} n_i=\varepsilon _{ij}\varepsilon _{bk}n_b n_j n_l\partial _l v_k + s^-_{ij}n_j +\varGamma h_i . \end{aligned}$$

The molecular field $\textbf{h}$ is defined in the standard way by Eq. (5) derived by varying the energy functional $\mathcal {F}= \int \mathcal {L}\textrm{d}\textbf{x}$. The Landau–de Gennes Lagrangian is expressed in 2D as

18 $$\begin{aligned} \mathcal {L} = \textstyle \frac{1}{2}K_1( \textrm{div}\, \textbf{n})^2 + \textstyle \frac{1}{2}K_2 ( \textrm{curl}\, \textbf{n})^2, \end{aligned}$$

where $K_1,K_2$ are splay and bend elasticities (Frank constants). This leads to the molecular field with the components

19 $$\begin{aligned} h_1&= K_1 (\partial ^2_1 n_1 +\partial _1\partial _2 n_2) +K_2(\partial ^2_2 n_1 -\partial _1\partial _2 n_2), \nonumber \\ h_2&= K_1 (\partial ^2_2 n_2 +\partial _1\partial _2 n_1) + K_2(\partial ^2_1 n_2 -\partial _1\partial _2 n_1). \end{aligned}$$

We expand the perturbation in the Fourier series $\widetilde{n}_i = \epsilon \int \widehat{n}_i (\textbf{k})\textrm{e}^{\textrm{i}\mathbf { k\cdot x}}\textrm{d}\textbf{k}$. Upon linearisation, the indices *b*, *j*, *l* in the first term on the right-hand side of Eq. (17) are set to 1, so that $i=k=2$ and only $n_2$ is coupled to hydrodynamics, with the product of the Levy-Civita symbols coming to $-1$ and $\partial _1 v_2=-\partial _1^2\varPsi$. Accordingly, the Fourier transform of Eq. (17) reads

20 $$\begin{aligned} \widehat{n}_1'(t)&= -\textstyle \frac{1}{2}k^2( K_1-K_2)\sin 2 \varphi \; \widehat{n}_2 , \end{aligned}$$

21 $$\begin{aligned} \widehat{n}_2'(t)&= -k^2 (K_1\sin ^2 \varphi + K_2\cos ^2 \varphi ) \widehat{n}_2\nonumber \\&\quad -k^2\left( \textstyle \frac{1}{2}+\cos ^2 \varphi \right) \widehat{\varPsi }. \end{aligned}$$

Stability is determined by the eigenvalues of the matrix $\textbf{J}$ defining the linearised dynamics of $\widehat{\varPsi },\,\widehat{n}_2$. Eq. (20) falls out, but in the case an instability develops, $n_1$ sets off as well, thereby changing the modulus and invalidating the director-based description. The elements of $\textbf{J}$ are

22 $$\begin{aligned} J_{\varPsi \varPsi }&=-\rho ^{-1}k^2\left( \eta _\textrm{s} +\eta _\textrm{a} \sin ^2\varphi \right) ,\nonumber \\ J_{\varPsi n}&= -\rho ^{-1} \zeta \sin 2\varphi , \quad J_{n\varPsi }=- k^2\left( \textstyle \frac{1}{2}+\cos ^2 \varphi \right) , \nonumber \\ J_{nn}&=- \varGamma k^2(K_1\sin ^2\varphi +K_2\cos ^2\varphi ) . \end{aligned}$$

The trace of $\textbf{J}$ is always negative, so that an oscillatory instability is excluded, and its determinant $\varDelta$ is

23 $$\begin{aligned} k^{-2} \rho \varDelta{} & {} =\zeta \cos 2\varphi \left( \textstyle \frac{1}{2}+\cos ^2 2\varphi \right) + k^2 \big [ \varGamma (K_1\sin ^2\varphi \nonumber \\{} & {} \qquad +K_2\cos ^2\varphi ) (\eta _\textrm{s} +\eta _\textrm{a} \sin ^2\varphi )\big ]. \end{aligned}$$

Activity dominates at long scales ($k \ll 1$), far exceeding the healing length, so that the absolute value of the instability threshold $|\zeta ^*|=\mathcal {O}(L^{-2})$ decreases with increasing system’s size *L*, and vanishes in an infinite system. Since the trigonometric function in Eq. (23) changes sign with $\varphi$, both extensile and contractile activity are destabilizing in alternative ranges of perturbation angles. In a finite system, the direction of the most dangerous perturbation also depends on the ratio of splay and bend elasticities, as well as on symmetric and antisymmetric viscosities and the shape of the enclosure.

### Vector-based stability analysis

A common deficiency of director-based theories is lack of conservation of the modulus $|\textbf{n}|=1$ in Eq. (13), made clear by taking its dot product with $\textbf{n}$. This feature is retained in Eq. (17) since, generally, $\mathbf {n\cdot h \ne 0}$, although, unlike Eq. (13), other terms do vanish there. A variable modulus $\varrho$ is commonly accounted for by taking as the order parameter a symmetric traceless matrix $\textbf{Q}$ with the elements $Q_{ij}=\varrho \left( n_in_j-\delta _{ij}/d\right)$, where $\varrho$ is the modulus and *d* is the number of dimensions [26]. In 2D, the more advantageous form of the order parameter, replacing $\textbf{Q}$, is the vector $\textbf{q}=\varrho \{\cos 2\theta , \sin 2\theta \}$, introduced as the *nemator* in Ref. [31] and independently applied by this author [29, 30]. The matrix with the elements $Q_{11}=-Q_{22}=q_1, \,Q_{12}=Q_{21}=q_2$ can be constructed by merging $\textbf{q}$ and $\varvec{\varepsilon } \cdot \textbf{q}$. The presence of the doubled inclination angle $\theta$ ensures the required invariance under rotation by $\pi$.

As before, we restrict to 2D, which suffices for stability analysis. Allowing for a variable modulus in vector nematodynamics based on Onsager’s reciprocal relations ensures long-scale stability in a passive system, but the full expressions are quite complicated and do not exclude a short scale instability on wavelengths approaching the healing length [29]. The cause of stabilisation lies in perturbations of the modulus coming with a lower degree of the wavenumber.

Similar to Sect. 3.1, the basic ordered state is $q_1=\varrho =1, \, q_2=0$, but, since the change of the modulus is allowed, there are now three coupled dynamic variables, as not only $q_2$ but also $q_1$ can be perturbed. The rigid rod model with the director-based equations (1)–(3) can be retained here. Rather than translating $\textbf{n}$ to $\textbf{q}$, which would involve clumsy trigonometry, these equations can be used directly for the purpose of linear stability analysis, but, since the nemator rotates twice faster than the director, both flow-dependent terms are doubled. Thus, Eq. (17) is modified, after replacing the indices $b,j,l \rightarrow 1$ in the first term as before, to

24 $$\begin{aligned} \mathcal {D} q_1=\varGamma h_1, \quad \mathcal {D} q_2=- 2 \partial _1 v_2 + 2 s^-_{21} +\varGamma h_2 . \end{aligned}$$

The Landau–de Gennes Lagrangian based on the vector $\textbf{q}$ is expressed in 2D as

25 $$\begin{aligned} \mathcal {L} = -\textstyle \frac{1}{2} \alpha _0\varrho ^2(1- \textstyle \frac{1}{2} \varrho ^2)+ \textstyle \frac{1}{2}K_1( \textrm{div}\, \textbf{q})^2 + \textstyle \frac{1}{2}K_2 ( \textrm{curl}\, \textbf{q})^2, \nonumber \\ \end{aligned}$$

This leads to the molecular field [29, 30]

26 $$\begin{aligned} \textbf{h}&=\textbf{q}(1-\varrho ^2) +\textbf{h}^\textrm{d}, \qquad h_k^\textrm{d}=\partial _i\pi _{ik}, \end{aligned}$$

27 $$\begin{aligned} h_1^\textrm{d}&= K_1 (\partial ^2_1 q_1 +\partial _1\partial _2 q_2) +K_2(\partial ^2_2 q_1 -\partial _1\partial _2 q_2), \nonumber \\ h_2^\textrm{d}&= K_1 (\partial ^2_2 q_2 +\partial _1\partial _2 q_1) + K_2(\partial ^2_1 q_2 -\partial _1\partial _2 q_1). \end{aligned}$$

The algebraic term in Eq. (26) is linearised as $-2\epsilon \widetilde{q}_1$, so that the Fourier transform of the perturbed Eq. (24) is expressed as

28 $$\begin{aligned} \mathcal {D} \widehat{q}_1&=-[2+k^2 (K_1\cos ^2 \varphi + K_2\sin ^2 \varphi ) ] \widehat{q}_1 \nonumber \\&\quad -\textstyle \frac{1}{2}k^2( K_1-K_2)\sin 2 \varphi \; \widehat{q}_2 , \nonumber \\ \mathcal {D}\widehat{q}_2&= -k^2 (K_1\sin ^2 \varphi + K_2\cos ^2 \varphi ) \widehat{q}_2 \nonumber \\&\quad -\textstyle \frac{1}{2}k^2( K_1-K_2)\sin 2 \varphi \; \widehat{q}_1 -k^2( 1+ 2\cos ^2 \varphi )\widehat{\varPsi }. \end{aligned}$$

The active stress is commonly defined as $\sigma _{ij}^\textrm{z}=-\zeta Q_{ij}$, which supplements the Fourier-transformed hydrodynamic equation (7) by the same term as in Eq. (16) with $\widehat{n}_i$ replaced by $\widehat{q}_i$.

The Jacobi matrix of the system (28) supplemented by the hydrodynamic equation is

29 $$\begin{aligned} \begin{pmatrix} -k^2\rho ^{-1}\left( \eta _\textrm{s}+\eta _\textrm{a} \sin ^2\varphi \right) &{} \quad \zeta \rho ^{-1}\sin 2\varphi &{}\quad - \zeta \rho ^{-1}\cos 2\varphi \\ 0 &{}\quad -2-k^2 H_{11}&{}\quad -k^2H_{12}\\ - k^2( 1+ 2\cos ^2 \varphi ) &{} \quad -k^2 H_{21} &{}\quad -k^2H_{22}, \end{pmatrix} \end{aligned}$$

where $H_{ij}$ are the elements of the matrix

30 $$\begin{aligned} \textbf{H}=\begin{pmatrix} (K_1\cos ^2 \varphi + K_2\sin ^2 \varphi ) &{}\quad \textstyle \frac{1}{2} ( K_1-K_2)\sin 2 \varphi \\ \textstyle \frac{1}{2}k^2( K_1-K_2)\sin 2 \varphi &{}\quad K_1\sin ^2 \varphi + K_2\cos ^2 \varphi \end{pmatrix}. \end{aligned}$$

Stability is determined by the signs of two Hurwitz determinants of the Jacobi matrix. The first Hurwitz determinant (the determinant of Eq, (29) with the inverted sign) defining the monotonic instability is computed as

31 $$\begin{aligned}{} & {} - k^{-2} \rho \varDelta \nonumber \\{} & {} \quad = -2 \zeta \cos 2\varphi \left( 1+2\cos ^2 2\varphi \right) + 2k^2 \varGamma \left( \eta _\textrm{s} +\eta _\textrm{a} \sin ^2\varphi \right) \nonumber \\{} & {} \qquad - k^2 \zeta \left[ 2H_{11}\cos ^3 2\varphi +H_{12}\sin 2\varphi \left( 1+\cos ^2 2\varphi \right) \right] \nonumber \\{} & {} \qquad + k^4\left( \eta _\textrm{s} +\eta _\textrm{a} \sin ^2\varphi \right) (H_{11}H_{22}-H_{12}H_{21}). \end{aligned}$$

The change of the modulus does not eliminate the domination of activity at long scales in this model, as the leading term in Eq. (23) differs from that in Eq. (31) just by the factor 2. An oscillatory instability is excluded here as well.

## Conclusion

The suggested mechanism incorporating odd and rotational viscosity, unusual in applications to media with nematic symmetry, may be only one of possible mechanisms responsible for interactions between flow and nematic order. The established way to derive these interactions through Onsager’s reciprocal relations, aspiring to be model-independent, turns out to be faulty as it allows for spurious instabilities. This may invalidate the results of certain derivations and simulations based on the established nematodynamic theory.

It is possible that no universal theory would be ever able to capture nematodynamic interactions in all applications and on scales ranging from molecular to macroscopic. Flow-alignment interactions and the way they are affected by activity are likely to be specific in different applications, especially biologically related, and require further deep insights. Activity, defined here in the standard manner, may be also introduced in different ways corresponding to specific mechanisms.

## Data Availability

No data are associated with the manuscript.
